# 

**Published:** 1863-01

**Authors:** 


### CONTENTS.

ORIGINAL ESSAYS AND COMMUNICATIONS.

Arsenic and its Compounds ........ 9
Success in the Dental Profession. By C. C. Dills, D. D. S. ........ 16
Metals and Alloys. By Dr. B. Wood ........ 23
Professional Etiquette. By W. H. Atkinson, D. D. S. ........ 31

SELECTIONS.

Dentition and its Derangements ........ 36
The Physical Knowledge ........ 40
Important India Rubber Case ........ 43

EDITORIAL.

Leeches Again ........ 50
Robertson's American Tooth Paste ........ 50
The Dentists Memorandum ........ 50
Mistakes of the Types ........ 51
Hudson's Tooth Paste ........ 51
Resignations ........ 51
Fang Filling ........ 52
Ohio Dental College Association ........ 53
Mississippi Valley Association ........ 53
Revival ........ 54
The People's Dental Journal ........ 54

